# Hospital Admissions for Respiratory Syncytial Virus (RSV) Among Elderly: A Retrospective Study From an Italian Southern Region During Years 2018–2023

**DOI:** 10.1111/irv.70315

**Published:** 2026-08-30

**Authors:** Giuseppe Di Martino, Pamela Di Giovanni, Federica Vaccaro, Francesca Romana Cascavilla, Carla Di Marzio, Livia Tognaccini, Edoardo Trebbi, Teresa Aita, Ferdinando Romano, Tommaso Staniscia

**Affiliations:** ^1^ Department of Medicine and Ageing Sciences “G. d'Annunzio” University of Chieti‐Pescara Chieti Italy; ^2^ Unit of Epidemiology and Health Statistics Local Health Authority of Pescara Pescara Italy; ^3^ Post‐Graduate School of Hygiene and Preventive Medicine “G. d'Annunzio” University of Chieti‐Pescara Chieti Italy; ^4^ School of Public Health “La Sapienza” University of Rome Rome Italy; ^5^ Local Health Authority of L'Aquila L'Aquila Italy; ^6^ Department of Public Health and Infectious Diseases “La Sapienza” University of Rome Rome Italy

**Keywords:** elderly, epidemiology, hospitalization, Italy, respiratory syncytial virus

## Abstract

**Introduction:**

Respiratory syncytial virus (RSV) is one of the most common causes of acute respiratory infections in both children and adults over 60. RSV can cause severe lower respiratory tract infections in older adults, leading to an increase of morbidity and mortality. The aim of this study was to assess the incidence of hospitalizations due to RSV infections in the Abruzzo region of Italy among patients aged over 60 years.

**Methods:**

A retrospective study was performed in the Abruzzo region, evaluating all hospitalizations performed during years 2018–2023. Age‐ and gender‐standardized hospitalization rates for RSV were calculated. Patients' comorbidities and hospitalization‐related outcomes were also assessed.

**Results:**

During 2018–2023, 42 RSV‐coded hospitalizations were identified among adults aged ≥ 60 years. An additional RSV‐attributable burden was estimated by applying a literature‐derived attributable fraction to hospitalizations for unspecified viral bronchiolitis and pneumonia, resulting in an estimated total of 67 RSV‐attributable admissions. Annual age‐ and sex‐standardized RSV‐coded hospitalization rates showed substantial year‐to‐year variability. Poisson regression with correction for overdispersion identified no statistically significant temporal trend during the study period (IRR per calendar year 1.08, 95% CI 0.86–1.34; *p* = 0.516). Among patients with RSV‐coded admissions, the median length of stay was 11 days (IQR 6–18), and four patients (9.52%) died during hospitalization.

**Conclusions:**

RSV was associated with a measurable burden of hospitalization among older adults in Abruzzo. However, substantial year‐to‐year variability and the small number of RSV‐coded admissions warrant cautious interpretation of temporal patterns. No statistically significant temporal trend was identified during the study period.

## 1 Introduction

Respiratory syncytial virus (RSV) is one of the most common causes of acute respiratory infections (ARIs) in both children and adults over 60 [1]. Although the effects of RSV on infants are well‐known and studied, the effects of its pathogenicity in adults and its associated economic burden have been more overlooked [2].

The understanding of the burden of RSV in adults remains relatively limited, as most studies conducted to date have limitations, including factors such as sampling methods, influenza‐like diagnostic criteria, insufficient study duration, and inadequate age stratification, all of which contribute to an underestimation of the severity of RSV in older adults [3].

RSV can cause severe lower respiratory tract infections (LRTIs) in older adults, leading to an increase of morbidity and mortality [4], especially in those who are frail and have risk factors and comorbidities [5]. RSV‐ARIs contribute to a rise in hospital admissions in older adults [6], particularly during the winter season.

RSV is highly contagious, with a basic reproductive number (R0) of 3 (±0.6) [7]. The circulation of RSV is highly correlated with seasonality and the climatic characteristics of the affected geographic area [8]. In countries with temperate climates, RSV circulates during the winter months, with peak activity observed between December and January [9]. In tropical regions, RSV outbreaks commonly occur during the hot, humid, and rainy summer period. Typically, one of the two genotypes (A and B) predominates in a given season [10], with either alternating or co‐circulating on an annual basis, although regional variations may be observed [11].

In Italy, the virus is most prevalent from November to March, with a peak in incidence occurring in December and January, partially according with the flu season [12].

RSV is transmitted through respiratory droplets and contaminated surfaces, typically presenting with mild, cold‐like symptoms [13]. Most healthy adults may be asymptomatic, and RSV infections may resolve spontaneously without the need for medical intervention [14]. However, in certain populations, including infants, older adults, and individuals with chronic conditions or compromised immune systems, RSV can lead to significant respiratory morbidity [15].

The aim of this study was to assess the burden and temporal patterns of RSV‐coded hospitalizations among adults aged ≥ 60 years in the Abruzzo region of Italy.

## 2 Materials and Methods

A retrospective observational study was performed in Abruzzo, a southern Italian region, which has a population of approximately 1.3 million people. Hospitalization data were collected from the hospital discharge records (HDRs) of the region, including all admissions from January 1, 2018, to December 31, 2023.

The HDRs contain various data regarding patients' demographic details and hospitalization, including age, gender, city of residence, marital status, and other information such as admission and discharge dates and the discharge mode, with death also included. Additionally, each record contained a diagnosis‐related group (DRG) code for classification of the admission, along with up to six diagnoses (one primary diagnosis and up to five comorbidities) and a maximum of six procedures or interventions that the patient underwent during the hospitalization.

Diagnoses and procedures were coded using the International Classification of Diseases, 9th Revision, Clinical Modification (ICD‐9‐CM) system, the National Center for Health Statistics (NCHS) and the Centers for Medicare and Medicaid Services, Atlanta, GA, USA.

In the study period, HDRs from patients aged 60 years or older were extracted. In selecting hospital admissions, RSV‐coded hospitalizations were identified when at least one of the following ICD‐9‐CM codes was recorded in either the principal diagnosis or any of the available secondary diagnosis fields: RSV acute bronchiolitis (466.11), RSV pneumonia (480.1), or RSV infection/positivity (079.6). Included patients were categorized into three groups based on the ICD‐9 codes used for their relative diagnoses: the first group included patients with a specific diagnosis of RSV (previously described), the second group with acute bronchiolitis by unspecified organism (466.19), and the third group with viral unspecified pneumonia (480.9). Because RSV may be under‐recorded in administrative hospital data, an additional RSV‐attributable burden was estimated among admissions coded as acute bronchiolitis due to unspecified organism (ICD‐9‐CM 466.19) and unspecified viral pneumonia (480.9). The primary estimate applied an attributable fraction of 9%, derived from previously published evidence. As this parameter was not estimated directly from the present study population, sensitivity analyses were performed using alternative literature‐supported attributable fractions to assess the robustness of the burden estimates [16].

Because the proportion of RSV infections potentially included within non‐specific respiratory diagnoses is uncertain, a sensitivity analysis was performed using alternative attributable fractions. The primary analysis retained the 9% attributable fraction used in the original analysis. Based on published evidence showing substantial variation in the proportion of RSV among respiratory infections in older adults, additional scenarios assuming attributable fractions of 5% and 15% were evaluated. These values were intended to represent plausible lower and upper scenarios rather than precise estimates for the present population.

The primary analysis retained the 9% attributable fraction used in the original analysis, which is consistent with previous estimates indicating that approximately 2%–9% of pneumonia cases among older adults may be attributable to RSV. Given the substantial heterogeneity reported in the literature, sensitivity analyses were additionally performed assuming attributable fractions of 5% and 15%. These values were selected to approximate the lower and upper bounds of a European meta‐analysis reporting an RSV proportion of 10% (95% CI 5%–16%) among ARI, influenza‐like illness, or community‐acquired pneumonia cases.

Previous evidence estimated that RSV accounted for approximately 2%–9% of pneumonia among older adults, whereas a more recent systematic review reported RSV proportions around 10% (95% CI 5%–16%) among adults aged ≥ 50 years with ARI, influenza‐like illness, or community‐acquired pneumonia in Europe. Annual population denominators were obtained from official resident population data for the Abruzzo region and were stratified by sex and age group (60–79 and ≥ 80 years). Annual hospitalization rates were calculated using the corresponding population for each study year. For estimates referring to the entire 2018–2023 study period, the denominator was expressed as population person‐years, calculated as the sum of the annual populations aged ≥ 60 years across the six study years.

To evaluate patients' characteristics, all comorbidities of each patient were extracted from the HDRs. In particular, all diagnoses included in the Charlson Comorbidity Index (CCI) were considered and extracted to describe patients' characteristics.

### 2.1 Statistical Analysis

Continuous variables were summarized as mean and standard deviation (SD) or median and interquartile range (IQR), according to their distribution. Categorical variables were reported as absolute frequencies and percentages. Annual RSV‐coded hospitalization rates were calculated per 100,000 population among adults aged ≥ 60 years. Rates were directly standardized by sex and age group (60–79 and ≥ 80 years), using the Abruzzo resident population in 2018 as the standard population. Annual numbers of RSV‐coded hospitalizations and their corresponding standardized rates were reported together with 95% confidence intervals.

Temporal changes in RSV‐coded hospitalization rates were assessed using a Poisson regression model, with the annual number of hospitalizations as the dependent variable and the corresponding resident population as the exposure. Calendar year was included as a continuous variable, and the model was adjusted for sex and age group. Because moderate overdispersion was observed, standard errors were corrected using the Pearson scale parameter. Results were expressed as incidence rate ratios (IRRs) with 95% confidence intervals. Given the exceptionally low number of RSV‐coded hospitalizations observed in 2018, a sensitivity analysis excluding 2018 was performed to assess the influence of this observation on the estimated temporal trend. In addition, a negative binomial regression model was fitted as a sensitivity analysis to evaluate the robustness of the results to potential overdispersion. A two‐sided *p*‐value of < 0.05 was considered statistically significant. Analyses were performed using Stata version 20 (StataCorp, College Station, TX, USA).

### 2.2 Ethics Statement

The study was conducted in conformity with the regulations on data management of the Regional Health Authority of Abruzzo and with the Italian Law on privacy (Art. 20–21 DL 196/2003), published in the Official Journal, n. 190, on August 14, 2004. The data were encrypted prior to the analysis at the regional statistical office, when each patient was assigned a unique identifier. The identifiers eliminated the possibility of tracing the patients' identities. According to Italian legislation, the use of administrative data does not require any written informed consent from patients.

## 3 Results

In the period from 2018 to 2023, 42 (13.13%) RSV‐related hospitalizations, 98 (30.63%) admissions for bronchiolitis due to unspecified microorganism, and 180 (56.25%) for unspecified viral pneumonia were recorded in the population aged 60 years or over in the Abruzzo region.

The mean age was 75.24 (±8.98) in patients with RSV infection, 77.43 (±9.23) in patients with other bronchiolitis, and 78.29 (±9.68) in patients with other pneumonia.

The median length of hospital stay in patients with RSV was 11 days (IQR 6–18), in patients with nonspecific bronchiolitis was 9 days (IQR 6–14), and in patients with nonspecific pneumonia was 10 days (IQR 6–15).

Within the study population, one patient (2.38%) with a confirmed diagnosis of RSV, one (1.02%) diagnosed with viral bronchiolitis due to an unspecified organism, and six (3.33%) with unspecified viral pneumonia required admission to the intensive care unit (ICU).

About discharge mode, among patients with RSV, 4 (9.52%) died, 27 (64.29%) were discharged home, and 11 (26.19%) had other discharge modes. Among patients with other 71 (72.45%) were discharged to home, and 24 (24.49%) had other discharge modes, and among patients with other pneumonia 33 (18.33%) died, 103 (57.22) were discharged to home, and 44 (24.44) had other discharge modes. Among the 42 RSV‐coded hospitalizations, four patients died during hospitalization, corresponding to an in‐hospital mortality of 9.52% (95% CI 2.7%–22.6%). ICU admission occurred in 2.38% of RSV‐coded hospitalizations (1/42), with a wide confidence interval reflecting the small number of events.

Regarding patients' comorbidities, in the group with specific RSV infection, 4 patients (9.52%) reported chronic heart failure, 11 patients (26.19%) reported chronic obstructive pulmonary disease (COPD), and 8 patients (19.05%) reported diabetes.

Among patients with unspecified bronchiolitis, 12 (12.24%) reported chronic heart failure, 8 (8.16%) reported cerebrovascular disease, 29 (29.59%) reported COPD, and 13 (13.27%) reported diabetes.

Among patients admitted for viral unspecified pneumonia, 10 (5.56%) patients reported dementia, 76 (42.22%) patients reported chronic heart failure, 13 (7.22%) patients reported cerebrovascular disease, 28 (15.56%) patients reported COPD, 28 (15.56%) patients reported diabetes, 26 (14.44%) patients reported renal failure, and 15 (8.33%) patients reported cancer. Patients' characteristics were reported in Table 1.

**TABLE 1:** Characteristics of patients hospitalized during the study period.

	RSV	%	Other viral bronchiolitis	%	Other viral pneumonia	%
Total	42	13.13	98	30.63	180	56.25
Gender						
Male	20	47.62	53	54.08	90	50
Female	22	52.38	45	45.92	90	50
Age						
Mean ± SD	75.24 ± 8.98		77.43 ± 9.23		78.29 ± 9.68	
> 80 years	15	35.71	41	41.84	93	51.67
LOS median (IQR)	11 (6–18)		9 (6–14)		10 (6–15)	
ICU	1	2.38	1	1.02	6	3.33
Discharge mode						
Death	4	9.52	3	3.06	33	18.33
Home	27	64.29	71	72.45	103	57.22
Others	11	26.19	24	24.49	44	24.44
Comorbidities						
Dementia	1	2.38	—	—	10	5.56
Myocardial infarction	1	2.38	—	—	6	3.33
Chronic heart failure	4	9.52	12	12.24	76	42.22
Vascular disease	—	—	2	2.04	3	1.67
Cerebral vascular disease	1	2.38	8	8.16	13	7.22
COPD	11	26.19	29	29.59	28	15.56
Rheumatic disease	1	2.38	2	2.04	1	0.56
Liver disease	1	2.38	3	3.06	4	2.22
Diabetes	8	19.05	13	13.27	28	15.56
Renal failure	3	7.14	3	3.06	26	14.44
Cancer	3	7.14	4	4.08	15	8.33

Abbreviations: COPD = chronic obstructive pulmonary disease; ICU = intensive care unit; IQR = interquartile range; LOS = length of stay; SD = standard deviation.

After applying the predefined attributable fraction to hospitalizations for non‐specific bronchiolitis and viral pneumonia, an additional 25 admissions were estimated to be attributable to RSV: specifically, in addition to 42 cases of RSV infection, using the primary attributable fraction of 9%, an additional 25 admissions were estimated to be attributable to RSV, resulting in an overall estimated burden of 67 admissions. In sensitivity analyses, assuming attributable fractions of 5% and 15% resulted in estimated overall burdens of approximately 56 and 84 RSV‐attributable admissions, respectively [17].

**FIGURE 1:**
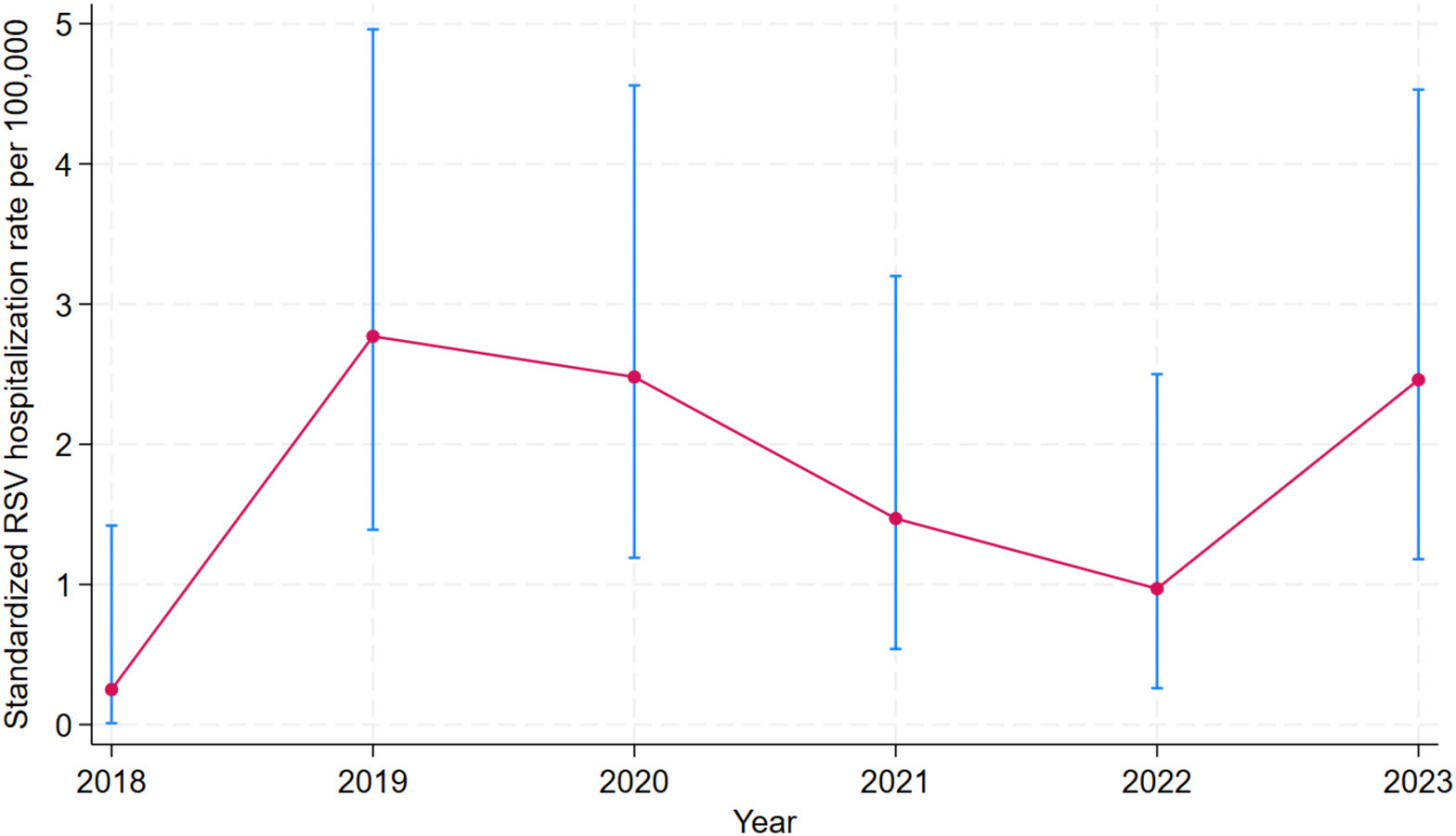
Annual age‐ and sex‐standardized RSV‐coded hospitalization rates among adults aged ≥ 60 years in Abruzzo, Italy, 2018–2023. Rates were directly standardized using the 2018 Abruzzo resident population as the standard population. Error bars represent 95% confidence intervals.

Figure 1 and Table 2 show trends in RSV‐related hospitalizations across the study period. Annual age‐ and sex‐standardized RSV‐coded hospitalization rates showed substantial year‐to‐year variability, ranging from 0.25 per 100,000 in 2018 to 2.77 per 100,000 in 2019. Rates were 2.48 per 100,000 in 2020, 1.47 in 2021, 0.97 in 2022, and 2.46 in 2023. Poisson regression with Pearson scale correction for overdispersion showed no statistically significant temporal trend in RSV‐coded hospitalization rates between 2018 and 2023 (IRR per calendar year 1.08, 95% CI 0.86–1.34; *p* = 0.516). Because only one RSV‐coded hospitalization was observed in 2018, a sensitivity analysis excluding this year was performed. The estimated direction of the temporal association reversed but remained statistically non‐significant (IRR per calendar year 0.90, 95% CI 0.72–1.12; *p* = 0.337), indicating that the temporal estimate was sensitive to the exceptionally low number of events recorded in 2018. A negative binomial sensitivity analysis produced consistent results (IRR 1.09, 95% CI 0.88–1.34; *p* = 0.440).

**TABLE 2:** Annual RSV‐coded hospitalization rates among adults aged ≥ 60 years in Abruzzo, 2018–2023.

Year	RSV‐coded admissions, *n*	Population ≥ 60	Directly standardized rate per 100,000	95% CI
2018	1	392,813	0.25	0.01–1.42
2019	11	396,979	2.77	1.39–4.96
2020	10	402,280	2.48	1.19–4.56
2021	6	405,197	1.47	0.54–3.20
2022	4	409,482	0.97	0.26–2.50
2023	10	413,959	2.46	1.18–4.53

Table 3 shows the hospitalization distribution among patients aged 60 years or older by the month of admission. In particular, a peak in RSV‐related hospital admissions was observed during the coldest season. Regarding viral bronchiolitis, the highest number of cases was recorded between February and May. The highest portion of other viral pneumonias occurred between January and April, except for a peak in August.

**TABLE 3:** Hospitalization distribution in patients > 60 years of age by admission month.

	RSV		Other viral bronchiolitis		Other viral pneumonia	
Month	*n*	%	*n*	%	*n*	%
January	14	33.33	8	8.16	17	9.44
February	6	14.29	12	12.24	20	11.11
March	5	11.90	10	10.20	29	16.11
April	5	11.90	10	10.20	20	11.11
May	0	0	10	10.20	12	6.67
June	1	2.38	4	4.08	12	6.67
July	1	2.38	4	4.08	11	6.11
August	0	0	4	4.08	15	8.33
September	1	2.38	7	7.14	6	3.33
October	1	2.38	11	11.22	12	6.67
November	3	7.14	9	9.18	11	6.11
December	5	11.90	9	9.18	15	8.33

## 4 Discussion

This study reported trends in RSV‐related hospitalizations among patients aged 60 years and over in the Abruzzo region, Italy, between 2018 and 2023, in a region with high circulation of this pathogen among infants [18].

This is the first and most recent study in Italy to consider RSV hospitalizations in adults over 60 years of age within the population of an entire region.

Indeed, there are limited studies on RSV in adults, and the burden of RSV in hospitalizations is underestimated, partly due to incorrect coding of the condition [19]. Moreover, the sensitivity of antigen‐based tests is low and inconsistent in adults, whereas other molecular methods, such as polymerase chain reaction (PCR), are more sensitive but significantly expensive [20].

A study conducted by the RSV Consortium in Europe (RESCEU) estimated that, based on data for children aged 0–4 years, 39% of the annual RSV‐associated hospitalizations in the EU occurred among persons aged ≥ 65 years [21]. Abruzzo region showed a lower proportion of RSV‐related admissions among elderly patients, but the present study was based on HDRs that could underestimate the real burden of disease.

An increment in the incidence of hospitalizations was observed over the analyzed period. Hospitalization rates varied considerably across individual years, particularly around the COVID‐19 pandemic period. However, these fluctuations should not be interpreted as distinct temporal phases, as no statistically significant overall temporal trend was identified. The observed annual fluctuations should be interpreted cautiously, particularly in light of the small number of events and the disruption of respiratory virus epidemiology during the COVID‐19 pandemic [22]. Considering all hospitalized patients, 2.5% required admission to ICUs. Regarding mortality, it was 12.50% of all hospitalizations, whereas the mortality rate specifically for RSV infections was 9.52%.

This study highlights that, according to the flu season, the peak in RSV‐related hospital admissions occurs during the coldest season, with a residual impact during the early spring months. For viral bronchiolitis, the highest number of admissions occurred between February and May, whereas the greatest number of cases of other viral pneumonias were recorded from January to April. However, there was an exception in August, when a peak was observed. The main reason for a different distribution of cases across months can be due to the different seasonality of the other pathogens than RSV.

These findings are consistent with previous evidence indicating that RSV may contribute to substantial morbidity among older adults, particularly among individuals with chronic conditions [23, 24].

The comorbidities most frequently observed in hospitalized patients were COPD, diabetes, and chronic heart failure, which increase the risk of ARI severity [25].

Until 2023, specific treatments and prevention strategies for RSV disease in older adults were limited; consequently, various vaccine and therapeutic candidates are under development [26]. Currently, two vaccines have been licensed for human use: Abrysvo from Pfizer Inc. (Pfizer Europe MA EEIG, Brussels, Belgium) and Arexvy from GlaxoSmithKline LLC (GlaxoSmithKline Biologicals SA, Rixensart, Belgium) [27].

According to literature, in the evaluation of a single‐dose vaccine efficacy against RSV‐associated LRTIs, characterized by the presence of three or more signs and symptoms, over two RSV seasons, Abrysvo exhibited an efficacy of 81.5% (95.0% CI, 63.3–91.6), whereas Arexvy demonstrated an efficacy of 67.2% (97.5% CI, 48.2–80.0) [28].

### 4.1 Strengths and Limitations

This paper presents some points of strength. First, it was the first evaluation of RSV‐related hospitalizations in adults over 60 years of age in the Abruzzo region, in a large study period (2018–2023), and including the full 2023 year. Poor studies were performed in Italy on this topic evaluating HDRs.

However, this study presents some limitations. First, the HDRs are used for admission‐related remuneration, so the evaluation of causes of admission could be underestimated due to miscoding. Second, the absence of important patient clinical data, such as drug therapies, blood parameters, and the clinical severity of each illness, influences the scope of our analysis. The use of a literature‐derived attributable fraction represents an important source of uncertainty. A recent systematic review and meta‐analysis of Italian adults showed considerable variability in RSV detection across studies and reported differences according to age, healthcare setting, study location, and sample size. Such heterogeneity suggests that a single attributable fraction may not be directly transferable across populations, healthcare settings, or surveillance systems [29]. Consequently, the 9% fraction used in the present study should be regarded as a modelling assumption rather than a population‐specific estimate. To address this uncertainty, we performed sensitivity analyses using alternative attributable fractions of 5% and 15%. The study period included the COVID‐19 pandemic, during which non‐pharmaceutical interventions, changes in healthcare utilization, and modified diagnostic testing practices substantially affected the circulation and detection of respiratory viruses. Consequently, temporal fluctuations observed during 2020–2022 may reflect both changes in RSV epidemiology and changes in case ascertainment. Finally, the use of HDRs focused only on hospitalized patients, lacking information on out‐of‐hospital RSV cases, leading to an underestimation of the burden of disease.

## 5 Conclusions

This study highlights the burden of RSV on hospital admissions among patients aged 60 years or over in a region of Southern Italy, showing that RSV represents an important cause of hospitalization. These results are consistent with previously published studies, highlighting the need for the implementation of effective preventive measures.

## Author Contributions

**Giuseppe Di Martino:** conceptualization, writing – original draft, methodology, formal analysis. **Pamela Di Giovanni:** conceptualization, software, data curation, validation. **Federica Vaccaro:** validation, visualization, writing – review and editing. **Francesca Romana Cascavilla:** methodology, data curation, investigation, writing – original draft. **Carla Di Marzio:** writing – original draft, investigation, methodology, data curation. **Livia Tognaccini:** investigation, validation, visualization, writing – review and editing. **Edoardo Trebbi:** investigation, validation, visualization, writing – review and editing. **Teresa Aita:** software, project administration, writing – review and editing, validation. **Ferdinando Romano:** conceptualization, validation, supervision, writing – review and editing. **Tommaso Staniscia:** conceptualization, methodology, supervision, resources, writing – review and editing, visualization, project administration.

## Ethics Statement

The study was conducted in conformity with the regulations on data management of the Regional Health Authority of Abruzzo and with the Italian Law on privacy (Art. 20–21 DL 196/2003), published in the Official Journal, n. 190, on August 14, 2004. The data were encrypted prior to the analysis at the regional statistical office, when each patient was assigned a unique identifier. The identifiers eliminated the possibility of tracing the patients' identities.

## Consent

According to Italian legislation, the use of administrative data does not require any written informed consent from patients.

## Conflicts of Interest

The authors declare no conflicts of interest.

## Data Availability

Data sharing is not applicable to this article as no datasets were generated or analyzed during the current study.
